# Amino Acids as Bio-Based Curing Agents for Epoxy Resin: Correlation of Network Structure and Mechanical Properties

**DOI:** 10.3390/polym15020385

**Published:** 2023-01-11

**Authors:** Florian Rothenhäusler, Holger Ruckdaeschel

**Affiliations:** 1 Department of Polymer Engineering, University of Bayreuth, Universitätsstraße 30, 95447 Bayreuth, Germany; 2 Neue Materialien Bayreuth GmbH, Gottlieb-Keim-Straße 60, 95448 Bayreuth, Germany

**Keywords:** amino acid, sustainability, bio-based, epoxy resin, mechanical properties, network structure, cross-link density

## Abstract

Bio-based alternatives for petroleum-based thermosets are crucial for implementing sustainable practices in fiber-reinforced polymer composites. Therefore, the mechanical properties of diglycidyl ether of bisphenol a (DGEBA) cured with either l-arginine, l-citrulline, γ-aminobutyric acid, l-glutamine, l-tryptophan, or l-tyrosine were investigated to determine the potential of amino acids as bio-based curing agents for epoxy resins. Depending on the curing agent, the glass transition temperature, Young’s modulus, tensile strength, and critical stress intensity factor range from 98.1$\;{}^{\circ }C$ to 188.3$\;{}^{\circ }C$, 2.6 GPa to 3.5 GPa, 39.4 MPa to 46.4 MPa, and 0.48 MPa m${}^{0.5}$ to 1.34 MPa m${}^{0.5}$, respectively. This shows that amino acids as curing agents for epoxy resins result in thermosets with a wide range of thermo-mechanical properties and that the choice of curing agent has significant influence on the thermoset’s properties. After collecting the results of dynamic mechanical analysis (DMA), tensile, flexural, compression, and compact tension tests, the functionality *f*, cross-link density $\nu_{C}$, glass transition temperature $T_{g}$, Young’s modulus $E_{T}$, compression yield strength $\sigma_{\mathrm{Cy}}$, critical stress intensity factor in mode I $K_{\mathrm{IC}}$, fracture energy $G_{\mathrm{IC}}$, and diameter of the plastic zone $d_{p}$ are correlated with one another to analyze their inter-dependencies. Here, the cross-link density correlates strongly positively with $T_{g}$, $E_{T}$, and $\sigma_{\mathrm{Cy}}$, and strongly negatively with $K_{\mathrm{IC}}$, $G_{\mathrm{IC}}$, and $d_{p}$. This shows that the cross-link density of DGEBA cured with amino acids has a crucial influence on their thermo-mechanical properties and that the thermosets considered may either be stiff and strong or tough, but hardly both at the same time.

## 1. Introduction

**Figure 1:**
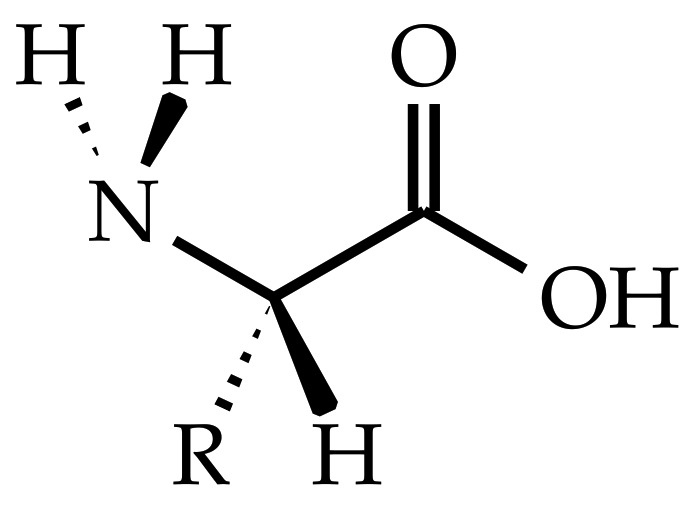
l-amino acid with its characteristic amino (-NH_2_) and carboxyl (-COOH) functional groups and the side chain (-R).

Fiber-reinforced polymer composites are commonly used in sports, automotive, wind energy, and aerospace due to their high weight-specific strength and modulus [1]. Here, epoxy resins are important matrix systems for fiber composites due to their high modulus and strength, high glass transition temperature, and low viscosity during fiber impregnation [2]. Commonly used curing agents for epoxy resins, such as amines [3,4], anhydrides [5] and phenolic compounds [6], are harmful in case of skin contact or ingestion. In contrast to that, amino acids are bio-based, biodegradable, and nontoxic compounds [7,8]. Amino acids are distinguished by their amino (-NH_2_) and carboxyl (-COOH) functional groups and a side chain (-R) that is unique for each amino acid (see Figure 1) [9]. Amino acids might be environmentally friendly alternatives for petroleum-based amines, as they have the same amino functional groups.

**Figure 2:**
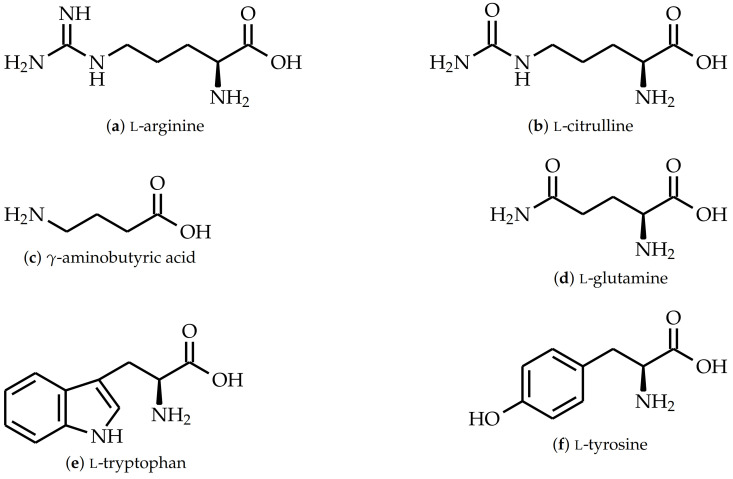
Chemical structures of amino acids used as curing agents.

Previous investigations on amino acid cured epoxy resins focused mainly on the reaction kinetics and glass transition temperature $T_{g}$ of l-tryptophan (see Figure 2e) and petroleum-based epoxy resins [10,11,12]. Depending on accelerator type and stoichiometric ratio, the $T_{g}$ ranges from 66 ${}^{\circ }C$ to 104 ${}^{\circ }C$ [13,14,15]. Contrary to that, Rothenhäusler et al. [16] investigated the glass transition temperature of DGEBA cured with l-arginine (see Figure 2a), which is about 100 ${}^{\circ }C$.

The tensile properties of an epoxidized sorbitol polyglycidyl ether cured with l-cysteine, l-arginine, or l-lysine was investigated by Shibata et al. [17]. Here, the tensile strength of the bio-based thermosets is 8.5 MPa, 10 MPa and 26.7 MPa, respectively. However, their tensile specimens (45 mm × 7 mm × 0.5 mm) were considerably smaller than the standardized 1B dog-bone specimens according to DIN EN ISO 527-2.

Rothenhäusler et al. [18] investigated the mechanical behavior of DGEBA cured with l-arginine at *T* = −40 ${}^{\circ }C$, 22 ${}^{\circ }C$, and 80 ${}^{\circ }C$. Here, the tensile, compression and flexural strength at 22 ${}^{\circ }C$ are about 56 MPa, 98 MPa and 85 MPa, respectively. Additionally, they found an intrinsic toughening effect caused by the finely dispersed amino acid particles which leads to a $K_{\mathrm{IC}}$ of about 1.1 MPa m${}^{0.5}$.

The thermal and mechanical properties of thermosets depend on their network structure, meaning the molecular structure of resin and curing agent, as well as the cross-link density $\nu_{C}$. Here, cyclic and aromatic structures lead to higher $T_{g}$, modulus, and strength, whereas aliphatic components reduce them [19,20,21]. However, aliphatic structures increase the mobility of network segments, which leads to increased toughness, i.e., increased $K_{\mathrm{IC}}$ and $G_{\mathrm{IC}}$ [19]. Typically, a high cross-link density is associated with high $T_{g}$, modulus, and strength, while the thermoset’s toughness decreases [22,23]. Since the thermal and mechanical properties of thermosets depend on the network structure, and as there are 22 proteinogenic α-amino acids that all possess distinct structures [8], it would be interesting to see the effect of structural differences in the curing agent on the thermoset’s properties.

Therefore, the objective of this investigation is to study the mechanical behavior of DGEBA cured with different amino acids. The goal is to determine the influence of the amino acid’s chemical structure on the tensile, flexural, compression, and fracture toughness properties of the thermoset and to determine the potential of different amino acids as curing agents for epoxy resins. After collecting the data of the mechanical characterization, key properties of the thermosets are correlated with one another to derive structure property relationships and obtain a general insight about the mechanics of thermosets.

## 2. Materials and Methods

### 2.1. Materials

D.E.R. 331 is a low-molecular-weight diclycidylether of bisphenol A (DGEBA) commonly used for prepegs with an epoxide equivalent weight (EEW) of 187 g mol${}^{-1}$ and viscosity of 12.5 Pa s at 25 ${}^{\circ }C$, and was purchased from Blue Cube Assets GmbH & Co. KG, Olin Epoxy (Stade, Germany). l-Arginine (purity 98.9%), l-citrulline, γ-aminobutyric acid (GABA) (purity 100%), l-glutamine (purity 100%), l-tryptophan (purity 100%), and l-tyrosine were purchased from Buxtrade GmbH (Buxtehude, Germany) and were provided as coarse powders. The side chain of each amino acid is different as they all possess different functional groups as well as a different number of carbon atoms between the α-amino group and the first functional group in the side chain (see Figure 2). Here, l-tryptophan and l-tyrosine have one carbon atom, l-glutamine has two carbon atoms, and l-arginine and l-citrulline have three carbon atoms between the α-amino group and their carbonyl group, amide group, or aromatic structures in the side chain. GABA has also three carbon atoms in its side chain; however, the amino group in GABA is bonded to the γ-carbon atom. 2-Ethyl-4-methyl-imidazole with a purity of 95% was bought from Tokyo Chemical Industry Co., Ltd. (Tokyo, Japan).

### 2.2. Resin Formulation

The preparation of the epoxy amino acid mixture follows the procedure already applied and described in [16]. Here, masterbatches with a stoichiometric ratio *R* equal to one of each amino acid with DGEBA were prepared via three-roll milling. Table 1 shows the assumed number of active hydrogen atoms in each amino acid and the resulting amine equivalent weight. Then, one weight percentage of the accelerator (2-ethyl-4-methyl-imidazole) was added before mixing with a BEVS 2501 series laboratory mixer from BEVS Industrial Co., Ltd. (Guangzhou, China) at 1000 min${}^{-1}$ for 15 min with a 30 mm mixing disk. Afterwards, the mixture was degassed for 60 min at 10 mbar to ensure the elimination of entrapped air prior to curing. The thermoset that results from curing the amino acid epoxy resin mixtures is referred to in this paper by the name of the amino acid that was used for curing the epoxy resin. For the sake of simplicity, the amino acid cured epoxy resins are referred to as amino-epoxides.

### 2.3. Curing Cycle and Sample Preparation

The amino acid epoxy mixture was poured into aluminum molds that were preheated at 60 ${}^{\circ }C$. The thermoset was cured in a Memmert ULE 400 convection oven from Memmert GmbH + Co. KG (Schwabach, Germany) for 2 h at 120 ${}^{\circ }C$ and 2 h at 170 ${}^{\circ }C$. The build-up of internal stresses was prevented by cooling down the molds to room temperature over 4 h. This curing cycle was already employed in the previous investigation about epoxy resins cured with amino acids and proved to be effective [18]. The specimens for dynamic mechanical analysis, compression tests, three-point bending tests, and compact tension tests were prepared with a Mutronic DIADISC5200 diamond plate saw from MUTRONIC Präzisionsgerätebau GmbH & Co. KG (Rieden am Forggensee, Germany) according to the test methods’ standards. The tensile specimens were CNC milled by a Mutronic Diadrive 2000 from MUTRONIC Präzisionsgerätebau GmbH & Co. KG (Rieden am Forggensee, Germany) according to the standards and their cutting edges ground manually with sandpaper with grain sizes from 30 μm to 10 μm.

### 2.4. Characterization Methods

#### 2.4.1. Dynamic Mechanical Analysis

The thermoelastic properties of the amino-epoxides were investigated via dynamic mechanical analysis on a Gabo Eplexor 500 N from Gabo Qualimeter Testanlagen GmbH (Ahlden, Germany) in tension mode. The specimens with dimensions 50 mm by 10 mm by 2 mm were measured from −120 ${}^{\circ }C$ to 240 ${}^{\circ }C$ with a constant heating rate of 3 K min${}^{-1}$. The tensile force amplitude was set to 60 N with a frequency of 1 Hz. Here, the glass transition temperature $T_{g}$ was taken as the temperature of the maximum value of the loss factor tanδ. The cross-link density of the thermoset $\nu_{C}$ in the rubbery state was calculated as

(1) $$\nu_{C}=\frac{E^{\prime }}{3RT}\;,$$

with the storage modulus $E^{\prime }$ at *T* = $T_{g}$ + 50 K and the universal gas constant *R* = 8.314 J mol${}^{-1}$K${}^{-1}$ [23]. For each thermoset, DMA was condicted three times.

#### 2.4.2. Tensile and Compression Tests

For the tensile tests, six 1B dog-bone specimens with dimensions 150 mm by 10 mm by 4 mm were tested with a cross-head speed of 5 mm min${}^{-1}$ according to DIN EN ISO 527-2. The thermosets’ compression modulus and strength were investigated according to EN ISO 604 on five and 12 specimens, respectively, with a cross-head speed of 5 mm min${}^{-1}$. Tensile and compression tests were carried out on a ZwickRoell Z020 universal testing machine from ZwickRoell GmbH & Co. KG (Ulm, Germany) using a load cell with a capacity of 20 kN.

#### 2.4.3. Three-Point Bending

For the three-point bending tests, 10 specimens with dimensions 80 mm by 10 mm by 4 mm of each thermoset were tested with a cross-head speed of 2 mm min${}^{-1}$ according to ISO 178. Three-point bending tests were carried out on a ZwickRoell Z020 universal testing machine from ZwickRoell GmbH & Co. KG (Ulm, Germany) using a load cell with a capacity of 20 kN.

#### 2.4.4. Fracture Toughness

The critical stress intensity factor in mode I $K_{\mathrm{IC}}$ and fracture energy $G_{\mathrm{IC}}$ were determined by testing ten compact tension specimens according to ISO 13586 on a ZWICK Z2.5 by ZwickRoell GmbH & Co. KG (Ulm, Germany) equipped with a load cell with a capacity of 2.5 kN. The fracture energy is calculated from $K_{\mathrm{IC}}$ via

(2) $$G_{\mathrm{IC}}=\frac{{K_{\mathrm{IC}}}^{2}}{E}\;\left(1-\nu^{2}\right)\;,$$

with Young’s modulus *E* taken from tensile tests and Poisson’s ratio ν, which is about 0.35 in the glassy state of the thermoset [24].

## 3. Results and Discussion

### 3.1. Dynamic Mechanical Analysis

**Figure 3:**
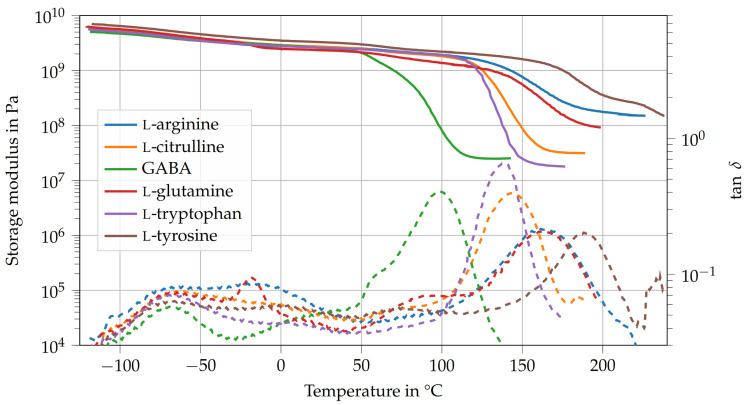
Dynamic mechanical analysis of amino-epoxides between *T* = −120$\;{}^{\circ }C$ and 240$\;{}^{\circ }C$.

Figure 3 shows the storage modulus $E^{\prime }$ and loss factor tanδ of the amino-epoxides. All thermosets possess a peak in tanδ at around −75 ${}^{\circ }C$. This could mean that the relaxation mechanism is related to the structure of DGEBA or some part of the molecular structure that all amino acids have in common. Usually, this β-relaxation is attributed to the hydroxy ether and diphenyl propane groups of DGEBA [20,25,26]. Additionally, l-glutamine shows a peak of tanδ at about −20 ${}^{\circ }C$. This relaxation maxima might result from the free rotation of the carbonyl group.

The thermosets’ $T_{g}$ (see Table 2) increase in the following order:

$$\begin{array}{c} T_{g,\mathrm{GABA}}<T_{g,L-\mathrm{tryptophan}}<T_{g,L-\mathrm{citrulline}}<T_{g,L-\mathrm{arginine}}<T_{g,L-\mathrm{glutamine}}<T_{g,L-\mathrm{tyrosine}} \end{array}$$

The relatively low $T_{g}$ of GABA (98.1 ${}^{\circ }C$) is the result of the low number of active hydrogen atoms (f=3), which leads to a low cross-link density $\nu_{c}$, combined with the long aliphatic side chain, which facilitates the rearrangement of network segments. Contrary to GABA, l-tryptophan possesses an indolyl group, a large aromatic double ring, which contains a secondary amino group, in its side chain. Here, there is only one carbon atom between the indolyl group and the α-amino group. However, the number of active hydrogen atoms is only four. The steric hindrance of the indolyl group might prevent the reaction between the secondary amine and the epoxy groups. All structural characteristics considered, the $T_{g}$ of l-tryptophan is about 40 ${}^{\circ }C$ higher (138.5 ${}^{\circ }C$) than that of GABA but still lower than that of the other amino-epoxides tested. l-citrulline has three carbon atoms in its aliphatic side chain but possesses more active hydrogen atoms (f=6) than GABA. Naturally, the increased cross-link density also leads to a higher glass transition temperature ($T_{g}$ = 143.5 ${}^{\circ }C$). The same holds true for l-arginine, which has an imine group instead of the carbonyl group in l-citrulline. This leads to an even higher cross-link density and an increase of $T_{g}$ of about 18 ${}^{\circ }C$ ($T_{g}$ = 161.8 ${}^{\circ }C$) compared to l-citrulline. The thermoset that was cured with l-glutamine has about the same $T_{g}$ (162.7 ${}^{\circ }C$) as l-arginine even though l-glutamine possesses fewer active hydrogen atoms (f=5) than l-arginine (f=7). It is likely that the reason for that is the structure of the amino acid’s side chain, as there are only two carbon atoms between the carbonyl group and the α-amino group. Thus, the shorter aliphatic chain poses a greater hindrance to rotations and rearrangements of network segments compared to the carbon chain in l-arginine. The amino-epoxy that used l-tyrosine as curing agent has a $T_{g}$ that is about 26 ${}^{\circ }C$ higher ($T_{g}$ = 188.3 ${}^{\circ }C$) than that of l-arginine and l-glutamine. Despite its low number of active hydrogen atoms (f=3), its glass transition temperature is higher than that of the other amino-epoxides tested or any $T_{g}$ of an amino-acid-cured epoxide reported in the literature [10,11,13,14,15,17,27]. Most notably, the $T_{g}$ of l-tyrosine is about 50 ${}^{\circ }C$ higher than that of l-tryptophan, even though l-tryptophan has more active hydrogen atoms while both possess aromatic side chains. One possible explanation for the high $T_{g}$ of l-tyrosine might be the possible reaction of its hydroxyl group and carboxylic acid to form an ester bond. This would lead to a highly cross-linked network with a substantial number of aromatic structures in each network segment. Additionally, l-tyrosine has only one carbon atom in its side chain apart from the phenyl group, which limits the rearrangement of network segments.

In general, the storage moduli of the amino-epoxides in the glassy state are similar to that of conventional epoxides. Here, the storage moduli of l-arginine, GABA, l-glutamine, and l-tryptophan are about 2.4 to 2.6 GPa. Contrary to that, l-citrulline and l-tyrosine possess slightly higher storage moduli than the other amino-epoxides, 2.8 GPa and 3.3 GPa, respectively. Interestingly, GABA and l-tyrosine, the amino-epoxides with the lowest and highest $T_{g}$, are also the ones with the lowest and highest storage moduli.

The cross-link densities of amino-epoxides range from 2400 mol m${}^{-3}$ for GABA to 13,450 mol m${}^{-3}$ for l-arginine. The wide range of possible glass transition temperatures, storage moduli, and cross-link densities of amino-epoxides shows that the type, quantity, and spatial arrangement of the curing agent’s functional groups are decisive for the thermoset’s thermo-mechanical properties.

In a previous investigation, Rothenhäusler et al. [18] characterized the mechanical properties of DGEBA cured with l-arginine, called Argopox, in the presence of a urea-based accelerator. Here, the thermoset’s $T_{g}$ was about 119 ${}^{\circ }C$, which is significantly lower than the $T_{g}$ of 162 ${}^{\circ }C$ that results from using 2-ethyl-4-methyl-imidazole as accelerator. Similarly, the cross-link density $\nu_{c}$ of the thermoset that uses 2-ethyl-4-methyl-imidazole as accelerator (13,450 mol m${}^{-3}$) is significantly higher than that of Argopox (2540 mol m${}^{-3}$). However, the storage modulus at room temperature is only slightly lower (2.6 GPa) than that of Argopox (2.7 GPa).

The $T_{g}$ of DGEBA cured with dicyandiamide ranges between 120 ${}^{\circ }C$ to 160 ${}^{\circ }C$, which is higher than the $T_{g}$ of GABA and lower than that of l-tyrosine [28,29,30]. The storage modulus and cross-link density of DGEBA cured with dicyandiamide is comparable to that of GABA or l-citrulline [31].

### 3.2. Tensile Tests

**Figure 4:**
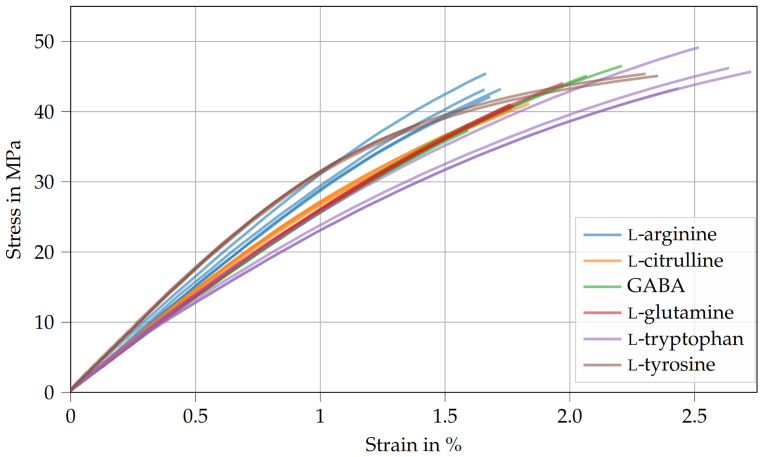
Stress–strain curves derived from tensile tests of amino-epoxides.

Table 3 shows the Young’s modulus $E_{T}$, tensile strength $\sigma_{T}$, and fracture strain $\varepsilon_{T}$ of the amino-epoxides. Here, the Young’s modulus ranges between 2.6 GPa and 3.5 GPa. The differences in Young’s moduli are similar to the differences in storage moduli observed during DMA (see Table 2). Interestingly, the tensile strength is about 40 MPa and is therefore more or less independent of the curing agent used (see Figure 4). Therefore, it is likely that the failure under tensile stress is caused by defects that are inherent to amino-epoxides. However, the investigation of the failure mechanisms is reserved for future studies. Similarly, there are only slight variations regarding the fracture strain (1.7% to 2.6%). Compared to typical epoxy resins cured with dicyandiamide, the tensile strength and fracture strain of amino-epoxides are significantly lower [32,33].

Notably, Young’s modulus, tensile strength, and fracture strain of Argopox are higher than that of DGEBA cured with l-arginine in the presence of an imidazole [18]. Imidazole accelerators also act as curing agents and promote the homopolymerization of epoxy groups [34], leading to an increased cross-link density. As a result, the thermoset becomes more brittle and is less able to tolerate stress concentrations caused by defects. Similarly to Argopox, the formation of water during the peptide reaction between amino acids or during the esterification of the amino acid’s carboxyl group with a hydroxyl group of DGEBA could lead to the formation of pores and, thus, lower the tensile strength [35,36,37].

### 3.3. Three-Point Bending

Table 4 shows the flexural modulus $E_{F}$, flexural strength $\sigma_{F}$, and fracture strain $\varepsilon_{F}$ of the amino-epoxides. Here, the flexural modulus ranges from 2.8 GPa to 3.7 GPa while the flexural strength of most of the amino-epoxides lies between 63 MPa to 71 MPa. This is similar to the results of the tensile test which showed that the tensile strength of amino-epoxides is virtually independent of the curing agent. Remarkably, the flexural strength (96 MPa) and fracture strain (4.4%) of the amino-epoxide cured with l-citrulline is considerably higher than that of the other thermosets. Therefore, the three-point bending of the thermoset cured with l-citrulline was repeated to ensure the results’ correctness. However, the second set of specimens showed no significant difference from the first set. Here, the flexural modulus and flexural strength are 3.3 GPa and 93.2 MPa, respectively. Thus, the investigation of this anomaly remains to be the focus of a future study. Compared to DGEBA cured with petroleum-based amines, the flexural strength of amino-epoxides is relatively low (95 MPa to 123 MPa) [20]. Similar to the tensile strength, the flexural strength of Argopox is higher than that of the l-arginine compound cured with the imidazole [18]. The reason for that might be the higher cross-link density caused by the imidazole.

### 3.4. Compression Tests

Table 5 shows the compression modulus $E_{C}$, compression yield strength ${}^{a}$ $\sigma_{\mathrm{Cy}}$, and compression yield strain $\varepsilon_{\mathrm{Cy}}$ of the amino-epoxides. The compression moduli are similar to the flexural moduli and range from 2.9 GPa to 3.6 GPa. Notably, the themosets cured with l-arginine or l-tyrosine show no distinct yield in their stress–strain curves. Therefore, the maximum compression stress is listed instead. Contrary to the tensile and flexural strength, the compression yield strength varies greatly for the different amino acids and ranges from 81 MPa to 132 MPa. Comparison of Table 2 and Table 5 shows that the amino-epoxides with a high cross-link density also have a high compression yield strength. Further correlations will be shown and discussed in Section 3.6. Here, the compression yield strength of l-citrulline is comparable to that of DGEBA cured with dicyandiamide in the presence of DYHARD^®^ UR500 (113.7 MPa) [33]. Interestingly, the compression yield strength and yield strain of the l-arginine thermoset cured with the imidazole are higher than that of Argopox (98 MPa and 8.5%) [18].

### 3.5. Fracture Toughness

Table 6 shows the critical stress intensity factor in mode I $K_{\mathrm{IC}}$, fracture energy $G_{\mathrm{IC}}$, and diameter of the plastic zone $d_{p}$ of the amino-epoxides. Interestingly, the $K_{\mathrm{IC}}$ and $G_{\mathrm{IC}}$ vary by a factor of about 2.8 and 8.1, respectively. Here, the thermosets with the highest and lowest cross-link density, l-arginine and l-tryptophan, also have the highest and lowest $K_{\mathrm{IC}}$ and $G_{\mathrm{IC}}$. Therefore, the intrinsic toughening by the amino acid particles results in an increased $K_{\mathrm{IC}}$ and $G_{\mathrm{IC}}$. However, further in-depth investigations are necessary for the exact determination of toughening mechanisms in each amino-epoxide. Usually, the $K_{\mathrm{IC}}$ of liquid epoxy resins cured with dicyandiamide is in the range of 0.6 to 0.7 MPa m${}^{0.5}$ [33,38,39]. The $K_{\mathrm{IC}}$ of Argopox is about 1.1 MPa m${}^{0.5}$ [18], which shows that the positive effect of the amino acid particles on the thermosets’ toughness is negated by the high cross-link density of l-arginine.

### 3.6. Correlations between Material Properties

**Figure 5:**
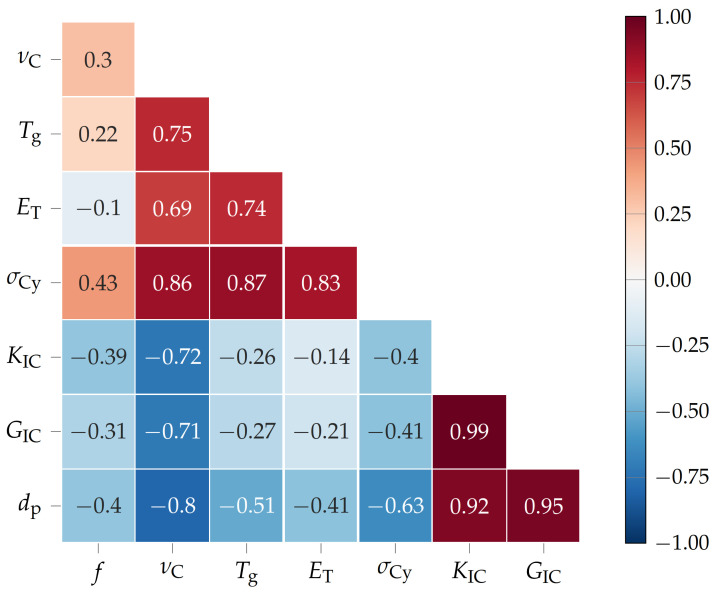
Pearson product moment correlation coefficients between functionality *f*, cross-link density $\nu_{C}$, glass transition temperature $T_{g}$, Young’s modulus $E_{T}$, compression yield strength $\sigma_{\mathrm{Cy}}$, critical stress intensity factor in mode I $K_{\mathrm{IC}}$, fracture energy $G_{\mathrm{IC}}$, and diameter of the plastic zone $d_{p}$ of the amino-epoxides.

After presenting the thermo-mechanical, tensile, flexural, compression, and fracture toughness properties of the amino-epoxides, it is time to discuss the interdependencies of key material properties. The Pearson product moment correlation coefficients *R* [40] (see Figure 5) of the functionality *f*, cross-link density $\nu_{C}$, glass transition temperature $T_{g}$, Young’s modulus $E_{T}$, compression yield strength $\sigma_{\mathrm{Cy}}$, critical stress intensity factor in mode I $K_{\mathrm{IC}}$, fracture energy $G_{\mathrm{IC}}$, and diameter of the plastic zone $d_{p}$ of the amino-epoxides were calculated via *numpy.corrcoef()* in Python 3.8.0 [41].

Firstly, the functionality *f*, meaning the number of active hydrogen atoms in the curing agent, i.e. the amino acid, correlates positively (*R* = 0.3) with the cross-link density $\nu_{C}$. Naturally, the more active hydrogen atoms the curing agent has, the more cross-links between DGEBA and curing agent that may form [21]. However, $\nu_{C}$ is also influenced by the molecular weight, meaning the size of the molecule that possesses the amino groups, of the curing agent. Therefore, the correlation between *f* and $\nu_{C}$ is rather weak. Interestingly, the $T_{g}$ correlates less strongly with *f* (*R* = 0.22), as a high $T_{g}$ can also be the result of the steric hindrance of aromatic structures, e.g. the indolyl and phenyl groups of l-tryptophan and l-tyrosine [20,42,43]. Since the Young’s modulus of thermosets in the glassy state at room temperature is, in general, about 3 GPa, there is only a weak correlation between *f* and $E_{T}$ (*R* = −0.1) [44]. Additionally, a high *f* correlates positively with compression yield strength $\sigma_{\mathrm{Cy}}$ (*R* = 0.43) because an increased *f* leads to a higher $\nu_{C}$ and therefore to a stiffer network [45]. Thus, the stress at which the thermoset starts to deform plastically is increased. Contrary to that, a high *f* correlates negatively with the critical stress intensity factor in mode I $K_{\mathrm{IC}}$ (*R* = −0.39), fracture energy $G_{\mathrm{IC}}$ (*R* = −0.31), and diameter of the plastic zone $d_{p}$ (*R* = −0.4). Because an increased functionality increases $\nu_{C}$, the thermosets become more brittle and their resistance against unstable crack propagation, as well as the fracture energy necessary for crack growth, is reduced [23,29,46].

Secondly, $\nu_{C}$ correlates positively with $T_{g}$ (*R* = 0.75) and $\sigma_{\mathrm{Cy}}$ (*R* = 0.86) as more cross-links make the network stiffer and impede the rearrangement of network segments [47]. Similar to *f*, $\nu_{C}$ correlates negatively but more strongly with $K_{\mathrm{IC}}$ (*R* = −0.72), $G_{\mathrm{IC}}$ (*R* = −0.71), and $d_{p}$ (*R* = −0.8). Consequently, there is a trade-off between high $T_{g}$, $E_{T}$, and $\sigma_{\mathrm{Cy}}$ on the one side or high $K_{\mathrm{IC}}$, $G_{\mathrm{IC}}$, and $d_{p}$ on the other side. This shows that these key properties can be tuned precisely by adjusting the cross-link density.

Next, the glass transition temperature $T_{g}$ correlates positively with $E_{T}$ (*R* = 0.74) and $\sigma_{\mathrm{Cy}}$ (*R* = 0.87) as all are influenced positively by stiff networks.

Lastly, the critical stress intensity factor in mode I $K_{\mathrm{IC}}$, fracture energy $G_{\mathrm{IC}}$, and diameter of the plastic zone $d_{p}$ all intercorrelate strongly (R> 0.9) with one another. Here, tough materials, meaning materials with high resistance to unstable crack propagation, also usually dissipate a lot of energy during crack growth. The energy dissipated during crack propagation is, of course, greater if the plastic zone at the crack tip, in which energy is dissipated during plastic deformation, is larger [33].

## 4. Conclusions

This study focused on the mechanical properties of DGEBA cured with either l-arginine, l-citrulline, γ-aminobutyric acid, l-glutamine, l-tryptophan, or l-tyrosine in the presence of an imidazole accelerator. Depending on the curing agent, meaning the amino acid, the glass transition temperature, Young’s modulus, tensile strength, and critical stress intensity factor range from 98.1 ${}^{\circ }C$ to 188.3 ${}^{\circ }C$, 2.6 GPa to 3.5 GPa, 39.4 MPa to 46.4 MPa, and 0.48 MPa m${}^{0.5}$ to 1.34 MPa m${}^{0.5}$, respectively. This shows that amino acids as curing agents for epoxy resins result in thermosets with a wide range of thermo-mechanical properties. However, the amino-epoxides reach performance levels that are lower than that of thermosets which are already used as matrix materials for fiber-reinforced composites.

The comparison between the mechanical performance of DGEBA cured with l-arginine in the presence of a urea or an imidazole shows that the imidazole accelerator leads to a higher cross-link density. From this follows that the imidazole increases the glass transition temperature, Young’s modulus, and compression yield strength, while the tensile strength, flexural strength, and fracture toughness properties are decreased considerably. Therefore, the choice of accelerator type is crucial during the design phase of thermosetting formulations.

The correlation of the functionality *f*, cross-link density $\nu_{C}$, glass transition temperature $T_{g}$, Young’s modulus $E_{T}$, compression yield strength $\sigma_{\mathrm{Cy}}$, critical stress intensity factor in mode I $K_{\mathrm{IC}}$, fracture energy $G_{\mathrm{IC}}$, and diameter of the plastic zone $d_{p}$ shows their interdependencies. Consequently, general statements about the nature of thermoset networks can be derived via analyzing strong correlations. Here, the cross-link density correlates strongly positively with $T_{g}$, $E_{T}$, and $\sigma_{\mathrm{Cy}}$, and strongly negatively with $K_{\mathrm{IC}}$, $G_{\mathrm{IC}}$, and $d_{p}$. This shows that the cross-link density of DGEBA cured with amino acids has a crucial influence on their thermo-mechanical properties and that the thermosets considered may either be stiff and strong or tough, but hardly both at the same time.

## Figures and Tables

**Table 1:** Molecular weight, assumed number of active hydrogen atoms (*f*), and resulting amine equivalent weight (AEW) of the amino acids used as curing agents.

Amino Acid	Molecular Weight in g mol${}^{-1}$	Functionality (*f*)	AEW in g mol${}^{-1}$
l-arginine	174.2	7	24.89
l-citrulline	175.2	6	29.20
GABA	103.1	3	34.37
l-glutamine	146.2	5	29.24
l-tryptophan	204.2	4	51.05
l-tyrosine	181.2	3	60.40

**Table 2:** Glass transition temperature $T_{g}$, storage modulus $E{}^{\prime }$, and cross-link density $\nu_{c}$ of amino-epoxides (average ± standard deviation).

Amino Acid	$T_{g}$ in ${}^{\circ }$C	E${}^{\prime }$ at *T* = 22 ${}^{\circ }$C in GPa	Cross-Link Density $\nu_{c}$ in mol m${}^{-3}$
l-arginine	161.8 ± 2.6	2.6 ± 0.1	13,450
l-citrulline	143.5 ± 0.7	2.8 ± 0.1	2730
GABA	98.1 ± 0.7	2.6 ± 0.1	2400
l-glutamine	162.7 ± 1.3	2.4 ± 0.0	7180
l-tryptophan	138.5 ± 0.7	2.6 ± 0.1	1560
l-tyrosine	188.3 ± 1.3	3.3 ± 0.0	11,770

**Table 3:** Young’s modulus $E_{T}$, tensile strength $\sigma_{T}$, and fracture strain $\varepsilon_{T}$ of amino-epoxides (average ± standard deviation).

Amino Acid	$E_{T}$ in GPa	$\sigma_{T}$ in MPa	$\varepsilon_{T}$ in %
l-arginine	3.0 ± 0.1	43.0 ± 2.1	1.67 ± 0.10
l-citrulline	2.9 ± 0.0	39.4 ± 2.5	1.69 ± 0.18
GABA	2.6 ± 0.0	41.4 ± 3.8	1.84 ± 0.25
l-glutamine	2.6 ± 0.1	43.5 ± 3.3	1.95 ± 0.25
l-tryptophan	2.8 ± 0.1	46.4 ± 1.9	2.57 ± 0.11
l-tyrosine	3.5 ± 0.1	42.8 ± 2.9	1.97 ± 0.40

**Table 4:** Flexural modulus $E_{F}$, flexural strength $\sigma_{F}$, and fracture strain $\varepsilon_{F}$ of amino-epoxides (average ± standard deviation).

Amino Acid	$E_{F}$ in GPa	$\sigma_{F}$ in MPa	$\varepsilon_{F}$ in %
l-arginine	3.2 ± 0.0	69.5 ± 5.9	2.34 ± 0.27
l-citrulline	3.1 ± 0.1	95.8 ± 10.5	4.43 ± 0.88
GABA	2.8 ± 0.0	66.9 ± 10.9	2.59 ± 0.6
l-glutamine	2.8 ± 0.0	62.9 ± 9.3	2.37 ± 0.54
l-tryptophan	2.9 ± 0.1	71.0 ± 4.0	2.94 ± 0.20
l-tyrosine	3.7 ± 0.1	70.8 ± 2.9	2.30 ± 0.15

**Table 5:** Compression modulus $E_{C}$, compression yield strength ${}^{a}$ $\sigma_{\mathrm{Cy}}$, and compression yield strain $\varepsilon_{\mathrm{Cy}}$ of amino-epoxides (average ± standard deviation).

Amino Acid	$E_{C}$ in GPa	$\sigma_{\mathrm{Cy}}$ in MPa	$\varepsilon_{\mathrm{Cy}}$ in %
l-arginine	3.3 ± 0.1	132.2 ± 12.2	23.4 ± 2.39
l-citrulline	3.2 ± 0.2	111.5 ± 11.3	12.2 ± 0.86
GABA	2.9 ± 0.2	80.8 ± 2.5	9.0 ± 0.54
l-glutamine	2.9 ± 0.1	103.3 ± 6.1	17.3 ± 2.41
l-tryptophan	3.0 ± 0.2	96.8 ± 6.4	11.2 ± 1.24
l-tyrosine	3.6 ± 0.2	132.2 ± 17.5	23.2 ± 3.76

${}^{a}$l-arginine and l-tyrosine do not show distinct yield behavior during compression, therefore the maximum compression stress and corresponding strain are listed instead.

**Table 6:** Critical stress intensity factor in mode I $K_{\mathrm{IC}}$, fracture energy $G_{\mathrm{IC}}$, and diameter of the plastic zone $d_{p}$ of amino-epoxides (average ± standard deviation).

Amino Acid	$K_{\mathrm{IC}}$ in MPa m${}^{0.5}$	$G_{\mathrm{IC}}$ in J m${}^{-2}$	$d_{p}$ in μm
l-arginine	0.48 ± 0.08	71 ± 24	1.4
l-citrulline	0.97 ± 0.09	288 ± 52	8.0
GABA	0.79 ± 0.15	219 ± 91	10.1
l-glutamine	0.64 ± 0.04	138 ± 17	4.1
l-tryptophan	1.34 ± 0.17	575 ± 146	20.3
l-tyrosine	0.82 ± 0.12	172 ± 54	4.1
